# A delayed and unsynchronized ovary development as revealed by transcriptome of brain and pituitary of *Coilia nasus*

**DOI:** 10.3389/fmolb.2024.1361386

**Published:** 2024-04-11

**Authors:** Ziyan Yu, Zongshuai Gao, Yun Zeng, Mingyou Li, Gangchun Xu, Mingchun Ren, Yunxia Zhu, Dong Liu

**Affiliations:** ^1^ Key Laboratory of Integrated Rice-Fish Farming, Ministry of Agriculture and Rural Affairs, Shanghai Ocean University, Shanghai, China; ^2^ Shanghai Universities Key Laboratory of Marine Animal Taxonomy and Evolution, Shanghai Ocean University, Shanghai, China; ^3^ Department of Transfusion Medicine, Shanghai Sixth People’s Hospital Afffiliated to Shanghai Jiao Tong University School of Medicinel, Shanghai, China; ^4^ Key Laboratory of Freshwater Fisheries and Germplasm Resources Utilization, Ministry of Agriculture, Freshwater Fisheries Research Center, Chinese Academy of Fishery Sciences, Wuxi, China; ^5^ Department of Laboratory Medicine, Shanghai East Hospital, Tongji University School of Medicine, Shanghai, China

**Keywords:** Coilia nasus, RNA-seq, gonad, pituitary, weighted gene co-expression analysis

## Abstract

Coilia nasus is an anadromous fish that has been successfully domesticated in the last decade due to its high economic value. The fish exhibits a delayed ovary development during the reproductive season, despite breeding and selection for five to six offspring. The molecular mechanism of the delayed ovary development is still unknown, so the obstacles have not been removed in the large-scale breeding program. This study aims to investigate the key genes regulating ovarian development by comparing the transcriptomes of ovarian-stage IV and stage II brain/pituitary of *Coilia nasus*. Ovarian stages were validated by histological sections. A total of 75,097,641 and 66,735,592 high-quality reads were obtained from brain and pituitary transcriptomes, respectively, and alternatively spliced transcripts associated with gonadal development were detected. Compared to ovarian Ⅱ- brain, 515 differentially expressed genes (DEGs) were upregulated and 535 DEGs were downregulated in ovarian Ⅳ- brain, whereas 470 DEGs were upregulated and 483 DEGs were downregulated in ovarian Ⅳ- pituitary compared to ovarian Ⅱ- pituitary. DEGs involved in hormone synthesis and secretion and in the GnRH signaling pathway were screened. Weighted gene co-expression network analysis identified gene co-expression modules that were positively correlated with ovarian phenotypic traits. The hub genes Smad4 and TRPC4 in the modules were co-expressed with DEGs including Kiss1 receptor and JUNB, suggesting that ovarian development is controlled by a hypothalamic-pituitary-gonadal axis. Our results have provided new insights that advance our understanding of the molecular mechanism of *C. nasus* reproductive functions and will be useful for future breeding.

## 1 Introduction

*Coilia nasus*, namely taper-tail anchovy, is a small-to medium-sized fish that can be divided into two stocks based on their life stages. One stock resides in the ocean and migrates to freshwater for reproduction, which is widely distributed in the coasts of the Northwest Pacific, including Korea, China, and Japan. In China, the migratory stock is mainly fished from the Yellow Sea and East China Sea (Ma et al., 2023). In spawning season, *Coilia nasus* adults aggregate in the estuary of the Yangtze River and then migrate to the middle and lower reaches of the Yangtze River for reproduction before their final gonadal maturation. The other is a freshwater-resident stock which spends its whole life cycle in the lakes in the lower reaches of the Yangtze River, and becomes the most dominant species in the lake ecosystem. It might be possible that chromosomal inversions caused the differences in morphology, physiology, and behaviour between the anadromous and resident stocks in the face of environmental heterogeneity in *C. nasus* (Zong et al., 2021). Over the past few decades, serious pollution, overfishing, and changes in aquatic ecology have resulted in a dramatic decline in populations of the migratory stock, which has a high economic importance in China (Zhang et al., 2005). However, *C*. *nasus* is highly responsive to stress, and this often causes death as soon as it is out of the water, which obstructed artificial breeding via capturing wild sexually mature individuals (Du et al., 2014). The Yangtze River to artificial pond diversion project was applied to capture wild juveniles of *C. nasus* (Zhang et al., 2006), therefore the tapertail anchovy was reared and domesticated in an artificial pond, and bred for five to six offspring to date.

The artificially reared taper-tail anchovy shows an evident phenomenon of population-asynchronous spawning, and the reproduction of tapertail anchovy continues to occur from April to July. Some three-year-old individuals have an asynchronous ovary development, although they breed in stable aquaculture conditions including nutrients, water temperature and environmental factors (Du et al., 2017). A best way to understand the mechanism of fish reproduction is to explore the gene regulation of ovarian development (Hayes, 1998). In teleost fish, reproduction is closely regulated by the hypothalamus-pituitary-gonadal (HPG) axis (Sofikitis et al., 2008). Gonadotrophin-releasing hormone (GnRH) in the hypothalamus, gonadotropin hormones (GHs) in the pituitary, and sex steroid hormones in the gonads have a pivotal role in the endocrine regulation of fish reproduction (Liu et al., 2013), resulting in complex reproduction modes, such as gonochorism (Jalabert, 2005), unisexuality (Schartl et al., 1995), and hermaphroditism (Avise and Mank, 2009). In general, fish sex determination is forced by genetic and/or environmental factors (Muralidhar and Veller, 2018; Bókony et al., 2019; Strüssmann et al., 2021). Genes in regulating sex determination and development exhibit spatio-temporal specificity in various species (Sinclair et al., 1990). Amh (anti-Müllerian hormone), Wnt (wingless-related integration site), and dmrt (Doublesex and mab-3-related transcription factor) have been identified to be involved in sex determination and sex differentiation processes (Dong et al., 2020; Farhadi et al., 2021; Liu et al., 2022). In addition, the biological signaling pathways are involved in steroidogenesis to mediate the gonad development of teleost fish, such as estrogen and TGF-β signaling pathway (Baroiller and Guiguen, 2001; Amberg et al., 2013). Recently data from taper-tail anchovy showed that several genes involved in the sex differentiation cascade exhibited characteristic expression in both gonadal somatic cell lines cultured *in vitro* (Kan et al., 2022) and in spermatogonial stem cell lines under long-term *in vitro* culture conditions (Gu et al., 2023).

RNA-seq technology has been widely used to profile genes involved in gonad development and their molecular mechanisms in fish (Du et al., 2017). Gender-specific transcriptomes have revealed a number of genes involved in gonad development, including medaka (*Oryzias latipes*) (Dechaud et al., 2021), threespine (*Gasterosteus aculeatus*) (Kaitetzidou et al., 2022), Amur sturgeon (*Acipenser schrenckii*) (Zhang et al., 2016), red-tail catfish (*Hemibagrus wyckioides*) (Wei et al., 2023) and *Coreoperca whiteheadi* (Liu et al., 2023). Transcript profiles were generated from sexually mature and immature gonads of fish species to identify sex-associated molecular markers (Amberg et al., 2013) and sex-related genes (Cao et al., 2022). High quality transcript assemblies were obtained from known reference genomes of the species. The whole genome sequences of taper-tail anchovy have been published (NCBI PRJNA422339) (Xu et al., 2020), and a gap-free reference genome sequence of anadromous *C. nasus* was available (SRP405363) (Ma et al., 2023). In the present study, gene expression profiles were first generated from the brain and pituitary tissues of female *C*. *nasus* at ovarian stages II and IV, respectively, under the same aquaculture conditions. Differentially expressed genes (DEGs) between ovary-stage II brain/pituitary and ovary-stage IV brain/pituitary were detected. Important candidate genes and pathways involved in ovary development were then identified. Weighted gene co-expression analysis (WGCNA) was comprehensively performed to discover the transcriptional network and the regulatory hub genes in response to ovary development. This study aims to detect gene coordination patterns in the hypothalamus-pituitary-gonad axis in *C. nasus* with unsynchronized ovary development and provides a theoretical basis for future artificial breeding.

## 2 Materials and methods

### 2.1 Experimental fish collection

Six healthy female *C. nasus* were collected simultaneously from Jiangzhiyuan Farm, Jiangsu Province, China, during the breeding season. Gonadal development was observed by anatomy and paraffin section, and three individuals with ovarian stage Ⅱ and three with stage Ⅳ were selected. Brain and pituitary samples were dissected and immediately stored in liquid nitrogen until the total RNA was extracted.

### 2.2 RNA extraction, library construction and sequencing

Total RNA was extracted using TRIzol reagent (Invitrogen, Carlsbad, CA, USA) according the manufacturer’s instructions. RNA concentration was determined using a NanoDrop-2000 spectrophotometer (Thermo Scientific, Waltham, MA, USA) and RNA integrity was determined by agarose gel electrophoresis. Poly(A) mRNA was isolated from the total RNA using Oligo (dT) and cleaved into short fragments using a Covaris S220 Focused-Ultra sonicator (Covaris, California, USA) and then used as templates for the synthesis of first- and second-strand cDNA according to the protocol of the Super Script Double-Stranded cDNA Synthesis Kit (Thermo Fisher Scientific, MA, USA). The cDNA was purified using a QIAquick PCR Purification Kit (QIAgen, Düsseldorf, Germany). After end repair, poly(A) addition and sequencing adapter ligation, the optional 300–400 bp fragments were selected by agarose gel electrophoresis, and enriched by PCR amplification to construct the cDNA library, followed by sequencing and generation of 150 bp paired-end reads using the Illumina HiSeq Ten platform. Three independent biological replicates were performed for stage Ⅱ and Ⅳ based on each tissue.

### 2.3 Read processing, reference sequence alignment, alternative splicing and novel transcript prediction

Raw reads were filtered using fastp v0.23.4 (https://github.com/OpenGene/fastp) to obtain high quality clean reads, and reads containing adaptor sequences, N% greater than 10%, and Q20% less than 20 were excluded. Clean reads were aligned to reference sequences (SRP405363) (Ma et al., 2023) using hisat2 (Shibaguchi and Yasutaka, 2022). The mapped results were used to determine alternative splicing occurring at different developmental stages using rMATs v4.0.2 (http://rnaseq-mats.sourceforge.net/rmats4.0.2/index. html). Transcripts for each sample were assembled using StringTie v2.2.2 (Pertea et al., 2015) and then merged into a set of transcripts for all samples to predict novel transcripts by comparison with annotation using gffcompare v0.11.2 (Pertea and Pertea, 2020).

### 2.4 Gene expression and differential expressed gene functional annotation

Gene expression counts were obtained from each sample via clean reads aligned to the reference using bowtie2 v2.5.2 (Mortazavi et al., 2008). Fragments per kilobase of transcript per million mapped reads (FPKM) values for the gene expression levels were calculated using eXpress v4.18.2 (Pertea et al., 2015). Relativity of biological replicates was calculated using the Pearson correlation coefficient between samples. Based on the gene expression level, differentially expressed genes were identified using DESeq2 v3 (Love et al., 2014). Thresholds for significant differential expression were set as a *p*-value <0.05, FDR (false discovery rate) ≤ 0.05, and absolute of log2 (fold change) ≥ 2. Finally, the differentially expressed genes were used for GO and KEGG enrichment analysis.

### 2.5 Identification of co-expressed network genes and hub genes

WGCNA analysis was used to assess the co-expressed gene clusters of brain and pituitary during the ovarian developmental periods using the OmicShare tools, a free online data analysis platform (https://www.omics.hare.com/tools). These expressed genes were clustered according to their log2 (FPKM) values. Pearson’s correlation coefficient was calculated for all genes obtained from brain and pituitary with ovarian developmental stage Ⅱ and stage Ⅳ, and a soft-threshold power (β = 8) was chosen to obtain a topological network. The hub genes in each module were identified by the value of kME (Module Eight Gene-based Connectivity).

### 2.6 qRT-PCR validation of differentially expressed genes

The qRT-PCR method was used to verify the differential expression of genes. Seventeen differentially expressed genes were selected from the brain and pituitary, and the β-actin gene was used as a control. The qRT-PCR primers were designed using the Primer Premier 6 (Bustin and Huggett, 2017), listed in Table 1, and synthesized by Sangon Biotech (Shanghai, China). The qRT-PCR was performed on an ABI 7500 Real-Time PCR System (ABI, New York, USA) using the 2^−ΔΔC^ method (Livak and Schmittgen, 2001). The reaction mixture consisted of 1 μL cDNA (60 ng/μL), 10 μL qPCR SYBR Green Master Mix (Hieff, Bioscience Inc., Hamburg, Germany), 0.5 μL of each primer, and 8 μL milli-Q water. Reactions were performed at 95°C for 30 s; 35 cycles of 95 °C for 5 s, 60°C for 20 s and 72°C for 10 s. The qRT-PCR results were obtained from three biological replicates.

**TABLE 1:** Sequences of primers used for gene differential expression analysis.

Gene	Tissues	Primer sequence (5->3)	
		Forward	Reverse
Hes5-1	Pituitary	CTG​TCA​ACT​TCC​TGT​CCC​AT	CTC​CAT​ATG​AAT​GTG​TGG​GTC
HES5-2	Pituitary	AGC​AGC​TCA​AGA​CTC​TAC​TGG	TGC​ACT​TGG​AGT​AGC​CCT​CA
HEY	Pituitary	TTT​CCC​CAG​CCA​CTA​CGG​ACA	TCT​CCA​GTT​TGG​CAG​AAC​CCT
HES5-3	Pituitary	ATG​ACC​TAC​TCA​ATG​GAG​CAT​C	TGC​ACT​TGG​AGT​AGC​CCT​CA
HES5-4	Pituitary	GAG​ACC​TGC​CAC​ATG​TCC​A	CTT​CTG​TCT​CAG​GAA​GCG​AAC
JUNB-1	Pituitary	GAC​CTT​CGC​CGA​GCC​ATA​CCG	TCC​TGC​TCC​TCC​GTG​ATG​CC
JUNB-2	Pituitary	AGC​TGT​TTT​ATC​ACG​ACG​ACT	TTG​TGA​TGA​CAC​CGT​TAC​CG
JUN	Pituitary	AGT​ACA​GCA​AAC​GCT​ATG​ACC	CTT​CCT​CTC​AGC​CTT​TAT​CCG
COX2	Pituitary	CAC​ACC​AGC​CTC​ATG​TTC​G	ATG​TAC​AAC​CTC​GCA​TCC​AC
SOCS3	Pituitary	AGA​AGG​TGC​CCC​ACT​TCG​ACT	GCA​CCT​TCT​CCC​CTC​CCG​AGT
MMP13-1	Brain	CTC​ATG​GGG​AAA​CAA​TCG​TCT	GCG​TCA​ATC​TTC​CTG​ATC​GTC​T
FOSB	Brain	GCT​CCT​CTC​CCT​CTT​TCG​AGT	TCA​CCG​TAG​GCA​CAA​ACG​AAC
CEBPB	Brain	CTG​GAT​TTG​ACA​TGC​GCT​CT	CGC​TGT​CCT​TAT​CCA​GAC​GTT
MMP13-2	Brain	CTC​TGT​TCT​TCA​CCG​GCA​AC	CTG​GTT​TCA​AGG​CTA​AAC​TCG
KISS1R	Pituitary	ACG​GCC​ACA​CTC​TAC​CCT​C	GCC​AAA​CCA​GTA​CCC​CGA​CT
Wts	Pituitary	CGC​GCC​ACC​CCA​CTA​CGA​CT	CCC​CGT​TCT​GCC​CTC​CGA​T
RNF151	Brain	TCA​GAG​CCA​CCG​CAC​CGT​TG	CTT​CTC​TCA​CTG​TCG​ATG​CCC​T
β-Actin		CAC​CAT​GTA​CCC​TGG​CAT​CG	TAC​TCC​TGC​TTG​CTG​ATC​CAC

## 3 Results

### 3.1 Histology of ovary

**FIGURE 1:**
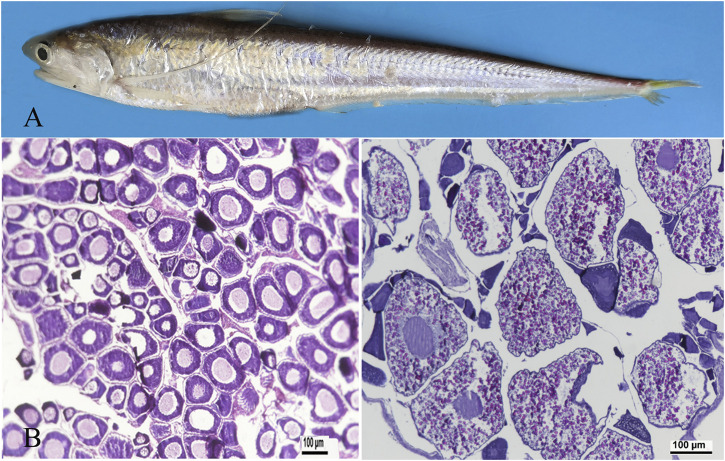
Representative photographs demonstrating ovarian developmental stages in adult taper-tail anchovy. **(A)** adult and female *Coilia nasus*. **(B)** ovary at stage Ⅱ. **(C)** ovary at stage Ⅳ. (Scale bar = 100 μm).

Ovaries were isolated from adult and 3-year-old females (Figure 1A) and fixed in Bouin’s fixative solution. The fixed tissues were embedded in paraffin and sections were taken from ovaries and stained with haematoxylin. Images were captured using a Zeiss Axio Imager M2 microscope (Zeiss, Canada). At ovarian stage Ⅱ, primary oocytes enter early growth, and exhibit early, middle, and late phase oogonium. The cytoplasm is highly basophilic and dark blue. Oocytes are 80–120 μm in diameter (Figure 1B). At ovarian stage Ⅳ, the ovarian cells are eosinophilic, and the yolk is highly concentrated. The yolk particles are round, filled with the whole cell and reddish. The diameter of the ovarian cells is 200–250 μm (Figure 1C).

### 3.2 Sequencing of *Coilia nasus* transcriptome

Transcriptome sequencing was performed on the six *C. nasus* individuals using Illumina HiSeq high-throughput sequencing, including six brain libraries and six pituitary libraries. After quality control, a total of 126.48 Gb of clean data was generated, and the Q30 base percentage in each sample was at least 94.38%. The clean data from the six *C. nasus* individuals are summarized in Table 2.

**TABLE 2:** Statistical analysis of sequence quality for *Coilia nasus* transcriptome.

Sample	Brain (ovary II)	Brain (ovary Ⅳ)	Pituitary (ovary II)	Pituitary (ovary Ⅳ)
Total bases	33277648500	34310229000	30130542300	29931490500
Clean bases	32990824434	33968236406	29909511658	2,9711964222
Clean reads	221850990	228734860	200870282	199543270
Total GC%	47.11	46.78	46.82	46.28
Mapped rate%	70.70	70.13	71.17	69.98

### 3.3 Alternative splicing

The alternative splicing events were analysed by rMATs software by comparing brain/pituitary at different stages of ovarian development using transcriptome assembly annotation (Supplementary Table S1), and five types of alternative splicing were detected, including exon skipping (ES), intron retention (IR), alternative 5′ splicing site (A5SS), alternative 3′ splicing site (A3SS) and mutually exclusive exon (MXE) (Table 3). Of these, seven alternative splicing transcripts were associated with sex-regulated and gonadal development (Table 4).

**TABLE 3:** Alternative splicing in brain/pituitary at different ovary stages.

Comparison	ES	IR	A3SS	A5SS	MXE
Brain at ovary II vs. IV	16,535	4,707	4,952	5,338	1707
Pituitary at ovary II vs. IV	15,770	4,583	4,759	5,198	1,575

**TABLE 4:** Alternative splicing of genes in regulating gonadal development.

Gene	Gene ID	Gene function	RPKM value			
			Brain (ovary Ⅱ)	Brain (ovary Ⅳ)	Pituitary (ovary Ⅱ)	Pituitary (ovary Ⅳ)
CYP17A	CectChr01G00296	Ovarian development	1.26	1.2	0.91	0.71
BDNF	CectChr05G03563	Gonadal development	37.46	28.83	37.14	29.8
IGF2	CectChr10G07823	Sex differentiation and gonad development	18.34	5.88	30.88	14.01
CTNNB1	CectChr04G02189	Gonadal development	40.01	11.19	82.43	20.17
DHH1	CectChr04G02189	Gonadal development	79.3	75.36	177.6	184.87
SMAD8	CectChr18G14984	Follicle development and ovulation	22.75	21.98	18.36	16.3
GNRHR	CectChr19G16830	Coding for gonadotropin-releasing hormone	1.27	2.11	3.5	3.14

### 3.4 Analysis of differentially expressed genes

**FIGURE 2:**
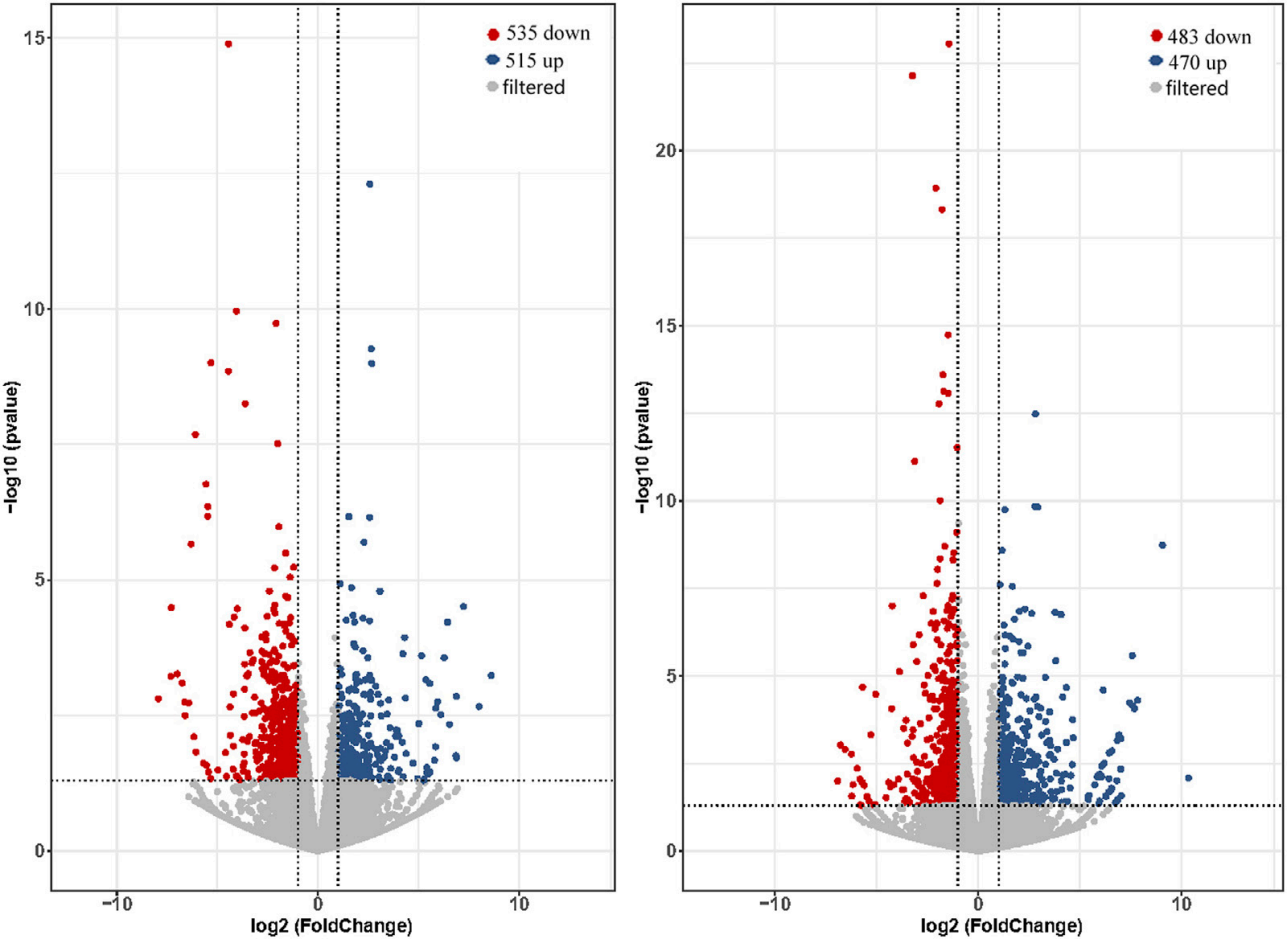
Volcano plot of DEGs calculated based on raw counts from brain (left) and pituitary (right) with ovarian development at stage Ⅳ *versus* stage Ⅱ.

The expression levels of total genes were calculated by FPKM values and were 26.13%–30.1% in low expression (0.1–3.75 for FPKM), 24.18%–30.30% in medium expression (3.75–15 for FPKM) and 31.84%–34.64% in high expression (>15 for FPKM). Comparing of brain and pituitary with ovary at different developmental stages, the ovary-stage Ⅳ brain showed upregulation of 515 DEGs and downregulation of 535 DEGs compared to ovary-stage Ⅱ brain. On the other hand, ovary-stage Ⅳ pituitary showed upregulation of 470 DEGs and downregulation of 483 DEGs compared to ovary-stage Ⅳ pituitary. Volcano plots were shown in Figure 2 (*p*-value <0.05 and absolute value of log2 (fold change) > 2).

### 3.5 DEG annotation and enrichment analysis

**FIGURE 3:**
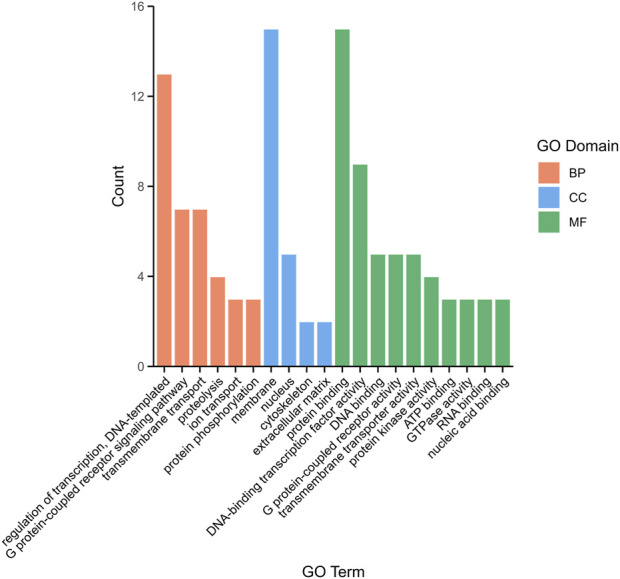
The top 20 distribution of DEGs among GO terms.

To explore the function of DEGs, a total of 1,085 comparative combination DEGs were obtained from brain and pituitary with unsynchronized ovary and subjected to Gene Ontology (GO) analysis. Three categories were annotated as being associated with binding in terms of biological process (BP, 37.65%), cellular component (CC, 19.43%) and molecular function (MF, 46.96%). Among the BP, regulation of transcription, DNA-templated (5.26%), G protein-coupled receptor signaling pathway (2.83%) and transmembrane transport (2.83%) were the most highly represented GO terms, whereas in the CC, membrane (6.07%) and nucleus (2.02%), and in the MF, protein binding (6.07%) and DNA-binding transcription factor activity (3.64%) (Figure 3).

**FIGURE 4:**
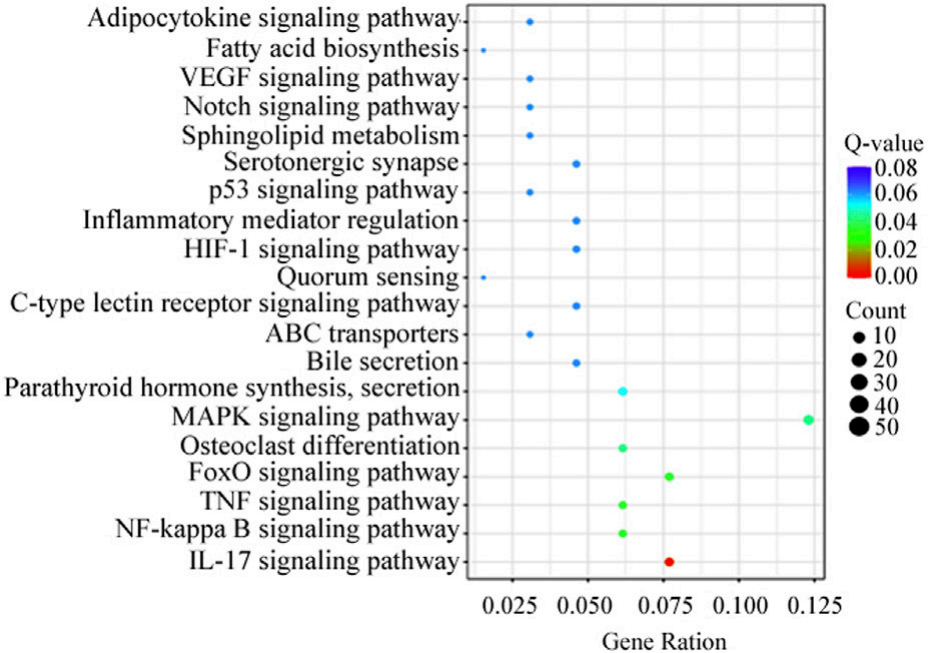
Top 20 distribution of DEGs presented in KEGG pathways.

KEGG analysis revealed that the DEGs were significantly enriched in 103 KEGG pathways. The top 20 were enriched in biological processes, including MAPK signaling pathway (12.3%), IL-17 signaling pathway (7.7%) and FoxO signaling pathway (7.7%) (Figure 4). Genes present in the KEGG pathways involved in hormone synthesis and secretion pathway to regulate ovary development via hypothalamus-pituitary-gonadal axis (Table 5).

**TABLE 5:** Gonad development-related DEGs in KEGG pathways.

Gene	Gene ID	Annotation	Brain pituitary			
			Log2 (Ⅳ/Ⅱ)	Qvalue	Log2 (Ⅳ/Ⅱ)	Qvalue
COX2	CectChr15G12738	Accelerated ovarian maturation			−2.785	0.017212
JUNB	CectChr19G16638	Gonadal development	−4.037	2.49E-08	−3.2401	1.23E-18
KISS1R	CectChr20G17331	Ovarian development			3.4737	0.024792
WTS	CectChr22G19126	Gonadal development			−2.1223	0.016587
RAS	CectChr19G15895	Ovarian development			−2.2335	3.27E-07
IGFBP1	CectChr24G21629	Sex differentiation and gonad development			−2.7716	0.0010136
SMAD9	CectChr17G13813	Follicle development and ovulation			−2.0318	0.010117
SLC14A	CectChr20G17313	Carrying neuropituitary hormones	−5.9747	0.0086992		
PAK6	CectChr22G19336	Interacting with androgen receptor	2.6848	1.75E-06		
HMR	CectChr14G11495	Mediates steroid synthesis	−2.1412	0.0072274	−2.5238	0.0058215
NOR1	CectChr21G18085	Mediates steroid synthesis			−2.4976	0.0012523

### 3.6 Validation of DEGs

**FIGURE 5:**
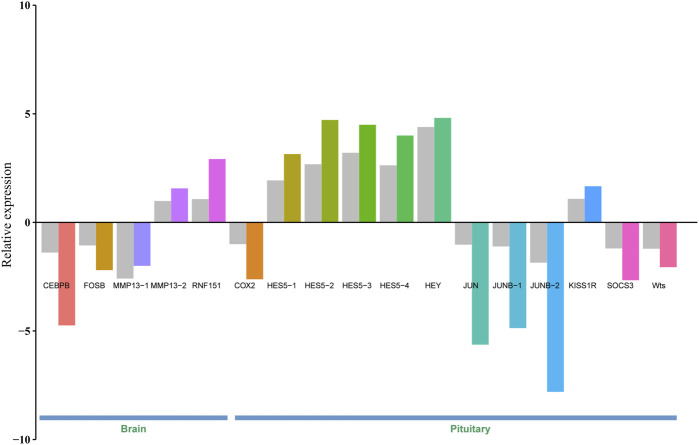
Validation of 17 DEGs in the brain and pituitary with ovary at stage Ⅱ and Ⅳ by qRT-PCR. Grey bar denotes FPKM value calculated by eXpress, and colour bar denotes qRT-PCR value.

To validate gene expression differences between brain/pituitary with ovary stage II and stage IV of *C*. *nasus*, 17 genes including CEBPB, FOSB, MMP13-1, MMP13-2, RNF151, COX2, HES5-1, HES5-2, HES5-3, HES5-4, HEY, JUN, JUNB-1 (jun B proto-oncogene 1), JUNB-2, KISS1R (Kiss1 receptor), SOCS3 and Wts were selected and applied a qRT-PCR method to determine their relative expression levels. Gene expression results from qRT-PCR were consistent with FPKM values from Illumina sequencing under the same conditions, indicating that DEGs from RNA-seq data were reliable (Figure 5).

### 3.7 Weighted gene co-expression analysis (WGCNA) of expressed genes

**FIGURE 6:**
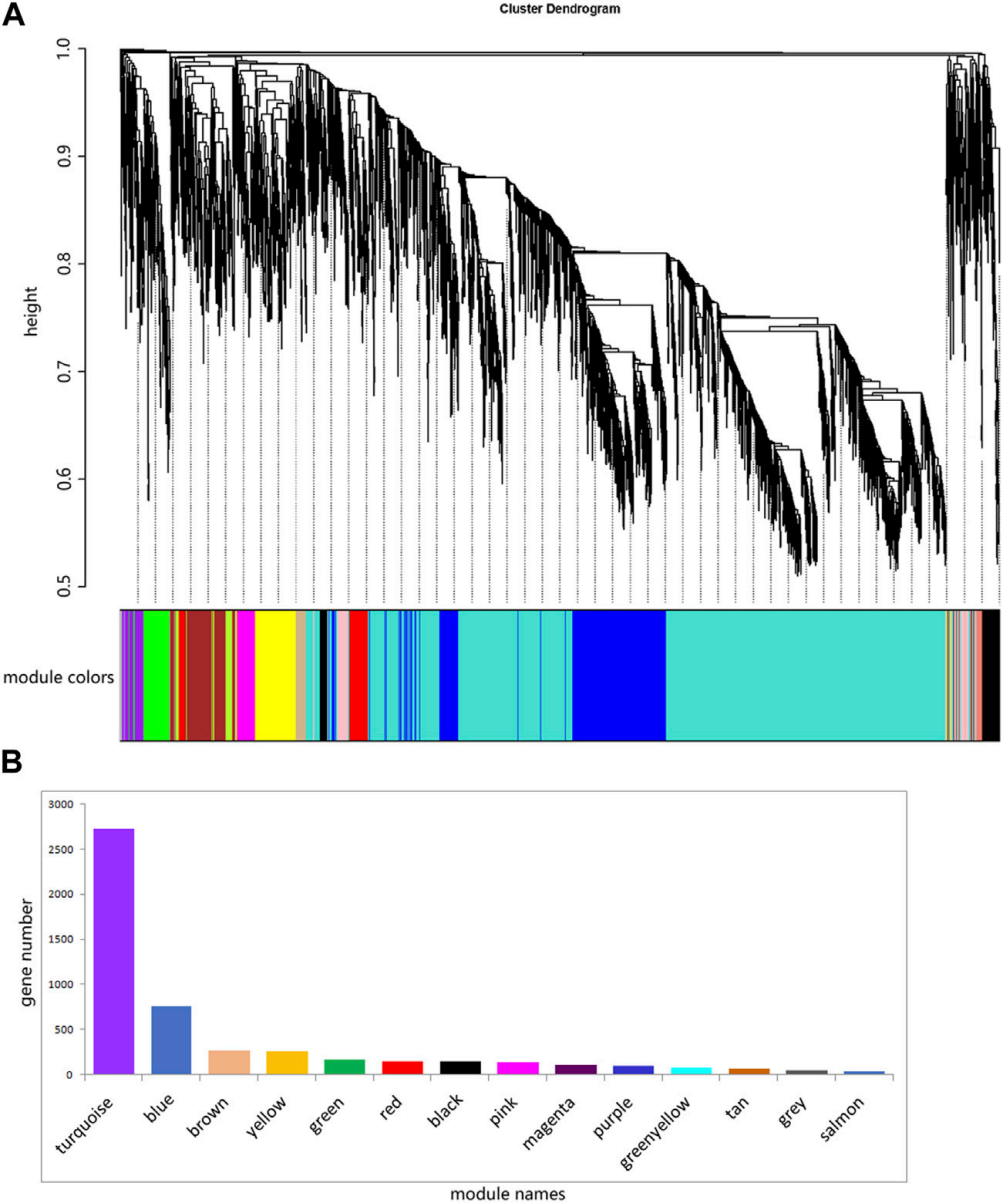
Fourteen different modules identified. **(A)** Gene co-expression network gene clustering number and modular cutting. The vertical distance of trees represents the distance between genes. Module colors are the module division of merged modules with similar expression patterns according to module similarity. **(B)** Number of genes per module. The abscissa represents each module, and the ordinate represents the number of genes.

**FIGURE 7:**
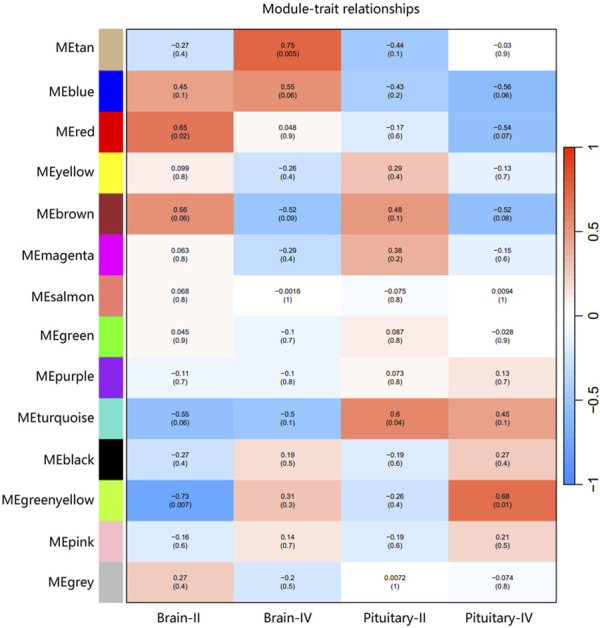
Correlation between gene co-expression network modules and brain/pituitary with ovary different traits. The horizontal axis represents different traits, and the vertical axis represents each module. The colour lattice and number without bracket represent a correlation between the ovary traits with the module, while the number with bracket represents a significant degree of correlation.

The ovary development process of *C. nasus* is controlled by interacting gene networks, and the weighted gene co-expression network analysis (WGCNA) method was applied to identify the gene modules with similar expression profiles during the ovary development periods of *C. nasus* and to analyze their biological function and metabolic pathways. As a result, 14 modules were obtained after dynamic cutting (Figure 6A). These modules had distinct gene sizes, ranging from 2,728 in the turquoise module to 30 in the salmon module (Figure 6B). Correlation analyses were performed between modules and brain/pituitary with ovary phenotypic traits at different developmental stages (Figure 7). The “red” and “tan” modules were significantly positively correlated (r ≥ 0.6, *p* < 0.05) with brain where ovary developmental traits were at stage II and IV, respectively. The “turquoise” and “greenyellow” modules were significantly positively correlated (r ≥ 0.6, *p* < 0.05) with pituitary, where ovary developmental traits were at stage II and IV stage, respectively.

### 3.8 Identification of hub genes in ovary stage-related modules

Hub genes were further identified in brain/pituitary modules with ovarian stage related traits. Genes with maximum abs (kME) value >0.9 and significance value <0.05 were selected as hub genes. A total of 47 (MEred), 12 (MEtan), 1,285 (ME turquoise) and 25 (ME greenyellow) hub genes were characterized in the four ovarian stage related modules (Figure 7). These modules were highly correlated with brain and pituitary with ovary stage traits (r ≥ 0.6). The key hub genes were screened out and evaluated based on the modules, including 1 gene regulating gonadotrophin secretion (Smad4, CectChr04G02957), 1 RNA-binding protein gene associated with brain developmental delay (R3HDM1, CectChr23G20501), 2 sensors of neurotransmission genes acting hypothalamic gonadotrophin-releasing hormone (TRPC4, CectChr17G13807; NHLH2, CectChr16G13558) and 1 gene regulating reproductive function (INSIG, CectChr06G04191).

**FIGURE 8:**
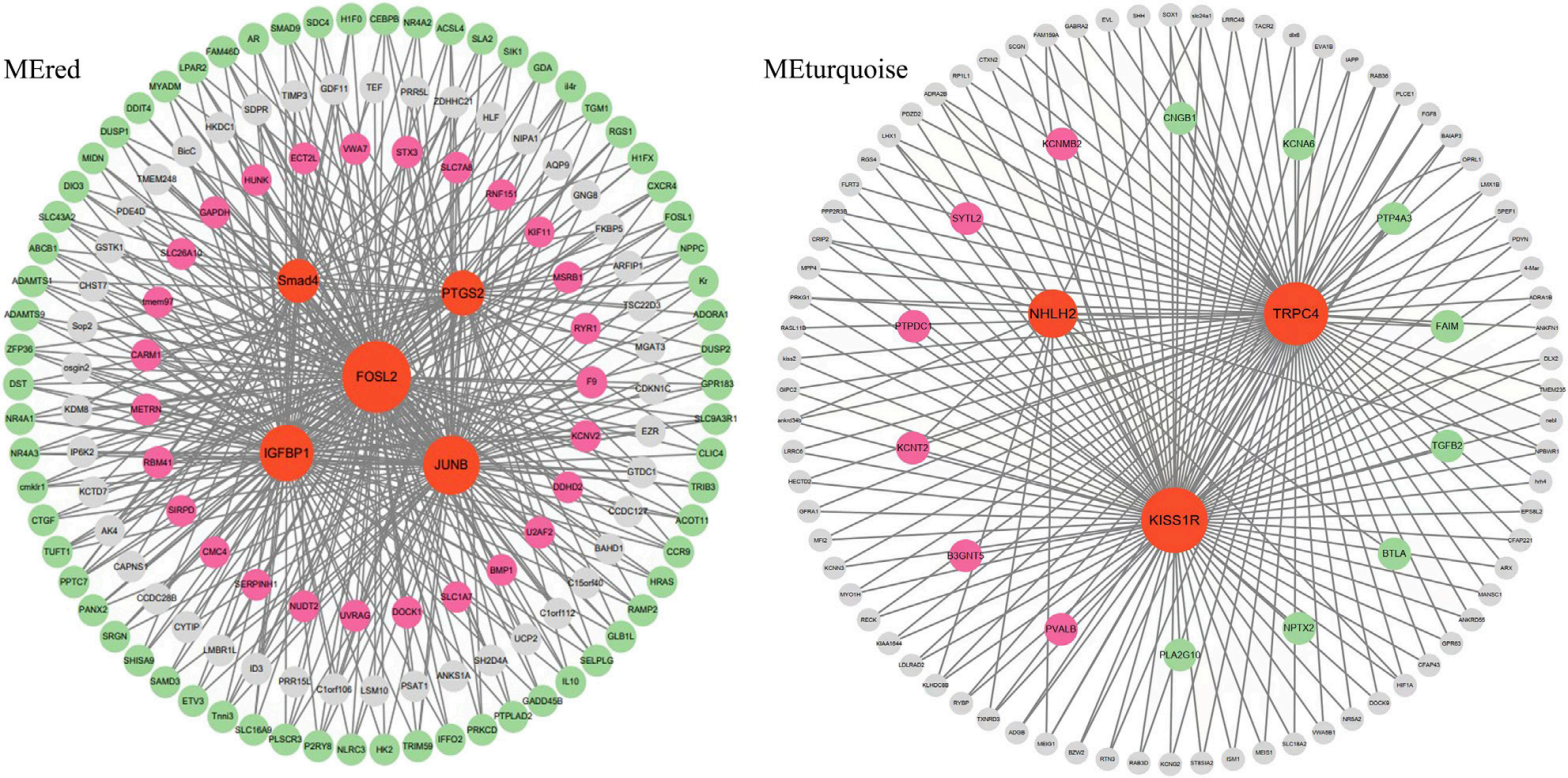
The network construction of hub genes in brain and pituitary with ovary at different development stages in two modules, MEred and MEturquoise.

Since Smad4, R3HDM1, TRPC4, NHLH2 and INSIG genes may play an important role in the brain-pituitary- gonadal axis, to further elucidate their functions in ovarian development, the co-expression gene networks of these genes were further analyzed. We found that Smad4 was co-expressed with DEGs including FOSL2, IGFBP1 and JUNB at the same time to form the main network. TRPC4, NHLH2 and KISS1R hub genes were co-expressed with either six up-expressed DEGs or eight down-expressed DEGs (Figure 8). R3HDM1 was co-expressed with two up- and six-expressed DEGs (Supplementary Figure S1). INSIG was co-expressed with 16 up- and 28 down-expressed DEGs (Supplementary Figure S1). These co-expressed hub genes were significantly enriched in signaling pathways related to ovarian development (Table 5), suggesting the reason for unsynchronized ovarian development leading to hormone release from the brain and pituitary.

## 4 Discussion

Transcriptome analysis is an effective approach to uncover molecular mechanisms at the gene expression level. In our study, a brain/pituitary transcriptome analysis was performed to characterize the unsynchronized ovarian development of *C. nasus* and to uncover reproductive mechanisms by regulating the brain-pituitary-gonadal axis (Kanda, 2019). In most teleosts, ovarian development can be classified as synchronous or asynchronous according to the growth pattern of oocytes (Scott, 1987). In synchronous spawners, a mature egg can be produced as an annual event over the lifetime of the individual. In contrast, in asynchronous spawners, mature eggs are ovulated in multiple batches from a developing oocyte during each spawning season. Xu et al. report that *C. nasus* females spawn once a year, and individual ovary development is significantly different in stage (the ratio of stage III: stage IV: stage V was 3:3:4) within a spawning season, despite *C. nasus* being artificially cultured with consistent environmental conditions (Xu et al., 2011). Xue et al. applied ovarian transcriptomic and metabolomic analyses to discover key signaling pathways, such as steroid hormone biosynthesis, that influence ovarian development in *C. nasus* (Xu et al., 2016).

For the anadromous *C. nasus*, the ovarian transcriptome profiles revealed a large proportion of genes involved in energy production, amino acid transport and metabolism during spawning migration (Duan et al., 2015) via food intake for energy consumption and energy storage (Yin et al., 2020). Brain transcriptome analyses of *C. nasus* from the Yangtze River showed that the brain plays a more regulatory role during migration, and the signal transduction pathways and genes relevant to neurotransmitter receptors, hormones and KISS1R were upregulated, providing key regulatory genes for gonadal development of anadromous *C. nasus* (Wang et al., 2020).

In our study, alternatively spliced transcript variants encoding different isoforms were found, and many gonad development-related transcript variants were detected in the *C. nasus* transcriptome data, including GnRHR (gonadotrophin-releasing hormone receptor), CYP17A1 (cytochrome P450 family 17 subfamily A 1), IGF-II (insulin-like growth factor II), and BDNF (brain-derived neurotrophic factor). GnRHR is mainly expressed in the pituitary gland and acts on the developing hypothalamic-pituitary-gonad axis by regulating sex hormone secretion (Li et al., 2022). CYP17A1 is a critical regulator of steroidogenesis in the conversion of pregnenolone to progesterone and, in the future, to 17-hydroxyprogesterone, which are processed to provide sex hormones. Mutant CYP17A1 resulted in abnormal sexual development (Singh et al., 2022). IGF-II is a reproduction-related growth factor, and can improve the developmental potential of oocytes, serving as a beneficial effect of growth factors in female reproduction (Muhammad et al., 2020). BDNF is heavily involved in the regulation of the hypothalamic-pituitary-adrenocortical axis (Naert et al., 2007). BDNF expression is correlated with gonadal hormones and its function is to regulate the products of gonadal steroids. Altered BDNF production and secretion has been implicated in a number of neurodegenerative diseases (Pluchino et al., 2013). These alternatively spliced transcripts obtained by comparing brain and pituitary at different stages of ovarian development suggested that alternatively spliced transcripts encoded by these genes may play an important role in reproduction of *C. nasus*.

Based on transcriptome data, DEGs affecting ovary growth were identified, including KISS1R, JUNB and IGFBP1. It was found that KISS1R, a key governor, provides the upstream signals for GnRH release, which subsequently regulates oocyte growth and maturation (Wang et al., 2011). Gonadotrophin hormones are glycoproteins produced by the pituitary gland that are required for normal reproductive function and gonadal development, and GnRH plays a fundamental role in the female reproductive system (Hollander-Cohen et al., 2021). Furthermore, KISS1R haploinsufficiency leads to premature ovarian failure, which is not rescued by ovarian gonadotrophin replacement (Gaytan et al., 2014). Interestingly, the kiss1 gene is absent in the gap-free genome of *C. nasus* (Gu et al., 2023), although JUNB showed significantly different expression in the brain and pituitary at different ovarian stages. JUNB was reported to be enriched within the Kiss1 promoter locus and showed a synergistic activation of Kiss1 promoter activity (Chakravarthi et al., 2018). IGFBP1 is a member of the soluble protein family, and either inhibits or stimulates the action of IGFs depending on the competition with IGF receptors for IGF-I, suggesting a potential role in the female reproductive ovary (Rutanen and Seppälä, 1992). The expression of the three genes was significantly different in brain and pituitary, and higher in pituitary with ovary at stage Ⅳ than Ⅱ for KISS1R, while lower in both brain and pituitary with ovary at stage Ⅳ than Ⅱ for JUNB and IGFBP1 in the ovarian development periods, and we speculate that the three genes have an important role in normal ovarian development.

WGCNA analysis of expressed genes identified 5 key hub genes from the “MEred”, “MEtan”, “MEturquoise” and “MEgreenyellow” modules. 1 gene regulating gonadotrophin secretion (Smad4), 1 brain developmental delay gene (R3HDM1), 2 genes acting hypothalamic gonadotrophin-releasing hormone (TRPC4, NHLH2) and 1 gene regulating reproductive function (INSIG) were important. These genes are involved in the brain-pituitary-gonadal axis, which is essential for ovarian development in fish. Ongaro et al. (Ongaro et al., 2020) reported that Smad4 stimulates the expression of follicle-stimulating hormone (FSH), an essential gonadotrophin hormone synthesized by pituitary gonadotrope cells. And the absence of Smad4 in gonadotropes renders females sterile (Fortin et al., 2014). The SMADs bind the FSH promoter in combination with the transcription factor Forkhead box L2, and an increase in FSH secretion is a key determinant of follicle maturation. In particular, there is an FSH threshold requirement for individual follicles, below which a given follicle will not develop (Odle and Childs, 2020). The hub genes in the “MEred” modules were more likely to be specific for ovarian development at stage Ⅳ, as four DEG hub genes (IGFBP1, FOSL2, JUNB and PTGS2) involved in fish reproduction were identified in these modules.

Meanwhile, the TRPC4 gene was a key candidate hub gene for ovarian development in *C. nasus*. TRPC4 is prominently expressed in the reproductive centre of the neuroendocrine brain, and TRPC4 activation contributes to kisspeptin to stimulate GnRH release (Götz et al., 2017). KISS1R is highly expressed in GnRH neurons and plays a crucial role in controlling pituitary gonadotrophin secretion and ultimately reproduction (Zhang et al., 2013). Network analysis of the hub gene TRPC4 found that the gene co-expressed with differentially expressed KISS1R gene were significantly function in governing GnRH neuronal excitability, which coincidentally regulate ovarian development in the reproductive axis in *C. nasus*, suggesting that they may co-regulate GnRH secretion in females, providing a plausible pathway for ovarian development at different stages.

## 5 Conclusion

In the present study, we detected 850/953 differentially expressed genes potentially associated with ovary development in the reproductive axis by transcriptome sequencing analysis of the brain/pituitary of *C. nasus* at different stages of ovary development. We identified 7 novel alternatively spliced transcripts, including CYP17A, IGF2 and GNRHR, that may affect ovarian development. In comparing the gene expression levels of brain/pituitary with ovary at differently developmental stages (stage Ⅳ vs stage Ⅱ) using RNA-seq, we identified 11 DEGS including KISS1R, IGFBP1 and WTS that may affect gonadotrophin secretion via regulation of the “hypothalamus-pituitary-gonad” axis. We also used WGCNA analysis to identify genes associated with gonadotrophin secretion in response to ovarian developmental traits. Four co-expression modules showed a high correlation with these ovarian stage traits, and the key hub genes Smad4, R3HDM1, TRPC4, NHLH2 and INSIG were screened out and their functions were involved in regulating ovarian tissue growth and development. Network analysis revealed that the key genes underlying these four modules were regulatory networks with DEGs. We provide a theoretical basis for delayed and unsynchronized ovarian development to improve artificial breeding of *C. nasus*.

## Data Availability

The datasets presented in this study can be found in online repositories. The names of the repository/repositories and accession number(s) can be found in the article/Supplementary Material.

## Supplementary material

SUPPLEMENTARY FIGURE S1 The network construction of hub genes in brain and pituitary with ovary at different development stages in MEtan and MEgreenyellow modules.

SUPPLEMENTARY TABLE S1 Transcriptome annotation for brain and pituitary of *Coilia nasus.*
