# Supplementary figures and images for: A delayed and unsynchronized ovary development as revealed by transcriptome of brain and pituitary of Coilia nasus
Source: Front Mol Biosci. 2024 Apr 11;11:1361386. doi: 10.3389/fmolb.2024.1361386 (PMC11043543; doi:10.3389/fmolb.2024.1361386)

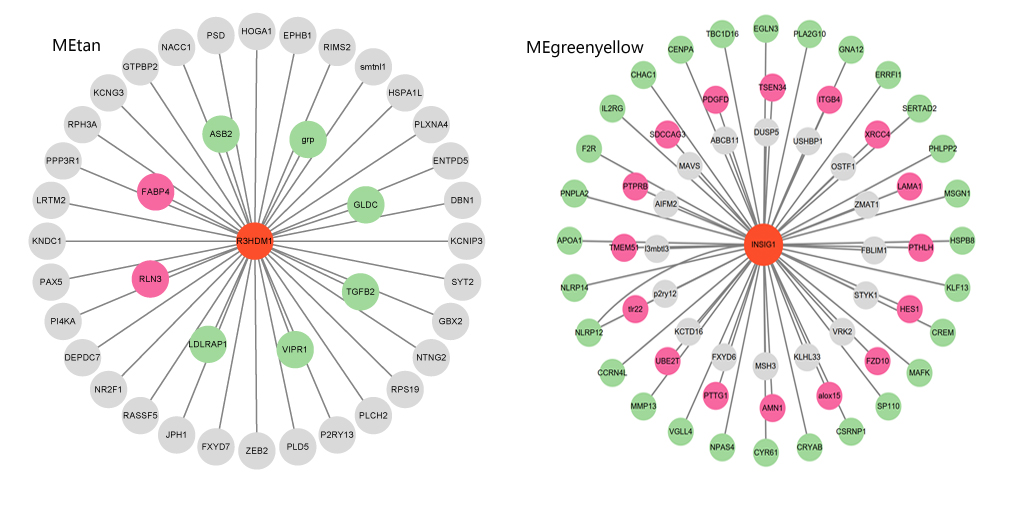

Supplement: Supplementary file 1 [file Image1.JPEG]
